# Interfacial Stabilization of Green and Food-Safe Emulsions through Complexation of Tannic Acid and Nanochitins

**DOI:** 10.1021/acsami.5c00132

**Published:** 2025-05-31

**Authors:** Qian Wu, Chen Zhou, Mengyao Niu, Jiaxin Hu, Nianjie Feng, Sameer Mhatre, Yi Lu, Xun Niu, Tianyu Guo, Jingqian Chen, Ran Bi, Orlando J. Rojas

**Affiliations:** † Hubei Key Laboratory of Industrial Microbiology, Key Laboratory of Fermentation Engineering (Ministry of Education), National “111” Center for Cellular Regulation and Molecular Pharmaceutics, 47896 Hubei University of Technology, Wuhan, Hubei 430068, China; ‡ Bioproducts Institute, Department of Chemical & Biological Engineering, 8166 The University of British Columbia, Vancouver, British Columbia V6T 1Z3, Canada; § School of Material Science & Chemical Engineering, Hubei University of Technology, Wuhan, Hubei 430068, China; ∥ Department of Chemistry, The University of British Columbia, 2036 Main Mall, Vancouver, British Columbia V6T 1Z1, Canada; ⊥ Department of Wood Science, The University of British Columbia, 2900-2424 Main Mall, Vancouver, British Columbia V6T 1Z4, Canada; 6 Department of Bioproducts and Biosystems, School of Chemical Engineering, Aalto University, FI-00076 Espoo, Finland

**Keywords:** chitin nanofibers, tannic acid, green emulsions, interfacial stabilization, food emulsions

## Abstract

Nanochitins exhibit unique structural attributes that confer distinct physical properties to multiphase systems. Amphiphilic tannic acid (TA) serves as an excellent candidate for interfacial modification via electrostatic adsorption and complexation with nanochitin. In this study, we developed green and food-safe strategies to enhance the stabilizing and functional performance of complexes formed through the coassembly of chitin nanofibers (ChNF) and TA. Their interactions were systematically investigated using spectroscopy, rheological measurements, and molecular simulations, all confirming strong interfacial binding primarily through hydrogen bonding. X-ray diffraction analysis further revealed TA-induced changes in ChNF crystallinity. The resulting ChNF–TA complexes effectively stabilized high internal phase Pickering emulsions (HIPPEs), which exhibited long-term stability and were successfully applied in direct ink writing. The exceptional stability of the HIPPEs was attributed to the synergistic effects of electrostatic charge neutralization and interfacial tension reduction. Quartz crystal microgravimetry demonstrated rapid complexation, with TA binding to ChNF thin films at a level of approximately 450 ng/cm^2^. The resulting HIPPEs remained stable for at least two months and readily formed cryogels upon freeze-drying. Owing to their enhanced stability and viscoelastic properties, HIPPEs stabilized with ChNF–TA complexes offer a promising platform for the development of sustainable emulsions, with the potential for customization in personalized food and related fields.

## Graphical abstract

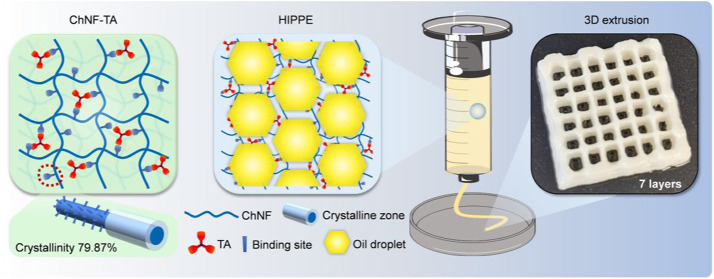

## 1 Introduction

Chitin is abundantly found in the exoskeletons of crustaceans and can be sustainably sourced from marine waste, particularly from the processing of crab, shrimp, and lobster shells, an underutilized biomass estimated at approximately 8 million tons per year globally.^1–3^ Composed of (1,4)-β-*N*-acetylglucosamine, chitin is a key polysaccharide precursor^4,5^ valued for its bioactivity, biocompatibility, noncytotoxicity, and environmental friendliness.^6–8^ In line with the growing interest in biologically derived nanomaterials, the potential of nanochitins has gained significant attention.^9,10^ Chitin nanofibers (ChNF) exhibit a high aspect ratio and are amphiphilic, while chitin nanocrystals (ChNC) display a rigid, rod-like morphology and higher crystallinity—attributes achieved through selective removal of chitin’s molecularly disordered regions. These unique physicochemical properties enable both ChNF and ChNC (herein collectively referred to as nanochitins) to act as effective stabilizers in multiphase systems, even at low concentrations.^11^

Emulsions are widely applied in the food, pharmaceutical, and cosmetics industries,^12–14^ where particle-stabilized (Pickering) systems are especially valued for their enhanced interfacial stability. A notable class within these systems is high internal phase Pickering emulsions (HIPPEs), characterized by an internal phase volume exceeding 74%. HIPPEs have found applications in food processing, such as sauces^15^ and dressings.^16^ Natural stabilizers like polysaccharides^17^ and proteins^18^ are often employed in food-grade emulsions. Chitin, owing to its cationic nature, readily forms electrostatic complexes with negatively charged molecules. Moreover, the degree of deacetylation can be adjusted to fine-tune its electrochemical properties,^20^ which in turn influence the structural and functional behavior of Pickering emulsions.

Polyphenols—abundant plant-derived compounds—are known to enhance emulsion stability.^21^ Tannic acid (TA), a naturally occurring polyphenol present in most plant tissues, is amphiphilic due to its multiple phenolic hydroxyl groups and aromatic rings.^22,23^ In addition to its anionic character, TA is a promising candidate for the interfacial modification of cationic nanochitins. Owing to its well-documented bioactivities, TA plays an important role in the nutraceutical and pharmaceutical sectors.^24^ The formation of electrostatic complexes between TA and nanochitins underpins their expected ability to form stable, functional Pickering emulsions.

In biological systems, chitin contributes to structural composites alongside proteins and minerals, forming exoskeletons that protect soft tissues from mechanical and environmental stresses.^25^ TA exhibits multifunctional roles, including antimicrobial and antioxidant effects, modulation of cell wall integrity through polysaccharide interactions, and mitigation of oxidative damage.^26^ Under suitable conditions, nanochitins form complexes with TA through electrostatic interactions, enabling them to act synergistically at the oil–water interfaces to stabilize multiphase systems. In this study, both ChNF and ChNC were evaluated based on their binding site characteristics, and gallic acid (GA) was employed as a reference molecule for TA.

This work investigates the interfacial stability and molecular mechanisms underlying the formation of ChNF–TA complexes. In addition, high internal phase Pickering emulsions (HIPPEs) stabilized by these complexes were utilized to develop three-dimensional-printed (3D-printed) structures and cryogels. The strategies presented herein represent a green and safe route for the formulation of emulsions suitable for food and pharmaceutical applications, while also laying a foundation for future research into advanced chitin-based technologies.

## 2 Results and Discussion

### 2.1 Chemical and Structural Changes upon Complexes Formation

#### 2.1.1 Chemical Fingerprints

The nanochitins (ChNF and ChNC) showed no significant absorption peaks in the 200–400 nm range, indicating the effective removal of proteins during their isolation (Figures 1a and S1a). We evaluated the pH changes in ChNF and ChNC suspensions and observed a decrease in pH upon the addition of tannic acid (TA) and gallic acid (GA) (Figure S2a). Furthermore, the absolute ζ-potential of ChNF decreased significantly upon the addition of TA, indicating strong electrostatic interactions. This effect was especially pronounced when compared with the other nanochitin–polyphenol complexes (Figure S2b).

**Figure 1:**
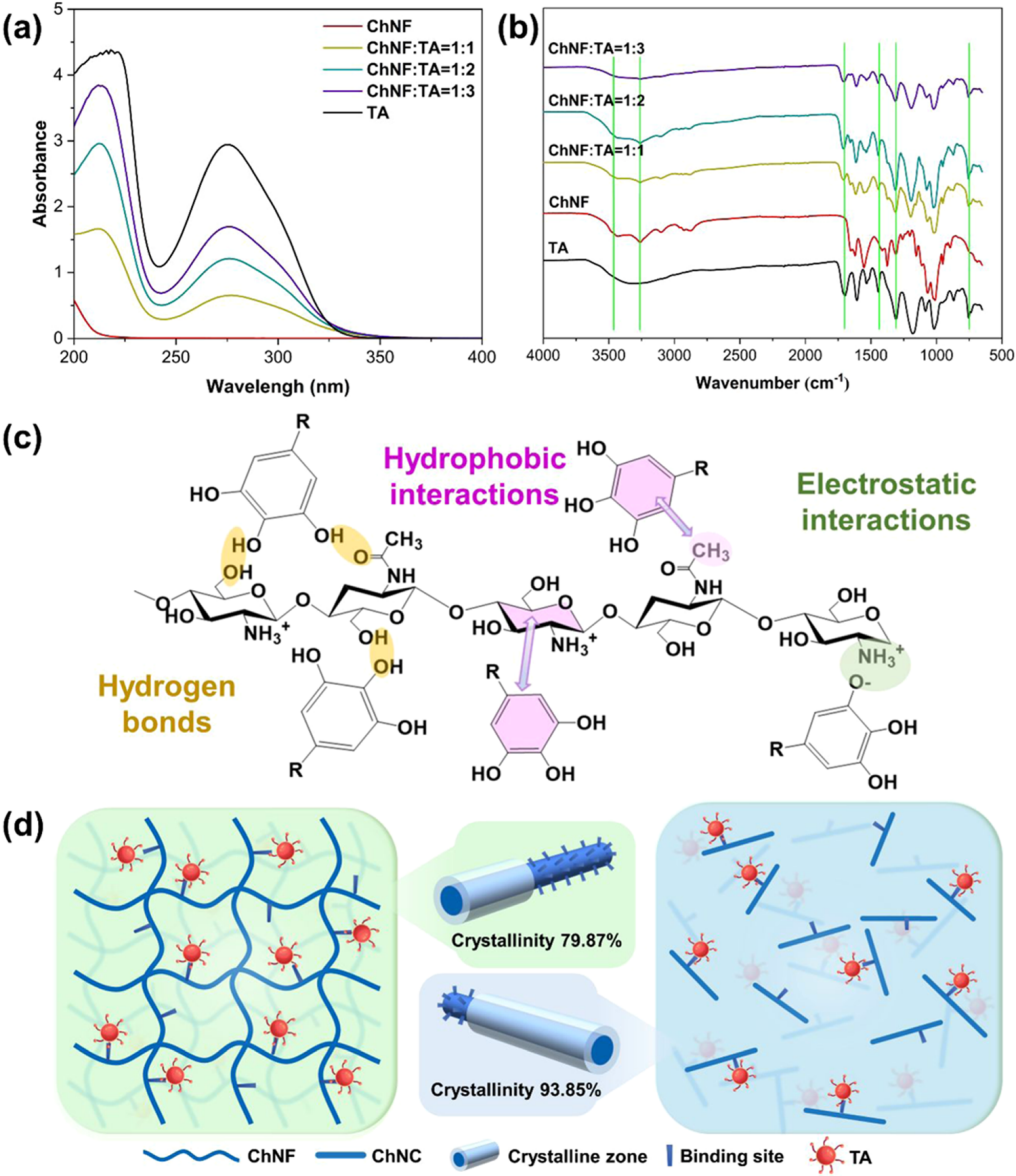
(a) UV and (b) FTIR spectra of ChNF-TA complexes. (c) Interactions between TA and nanochitins and (d) the respective schematic illustration for ChNF (left) and ChNC (right).

Fourier transform infrared spectroscopy (FTIR) was used to examine the interactions between ChNF and TA (Figure 1b). An increase in the intensity of the peak at 3340 cm^–1^ with increasing TA content indicates the formation of hydrogen bonds between ChNF and TA.^27^ Additionally, a shift in the peak position suggests the presence of hydrophobic effects between the two components (Figure 1c). Similar spectral changes were observed in the FTIR spectra of the other three complexes, although the intensity and peak shifts were less pronounced compared to those of the ChNF–TA complex (Figure S1b). Overall, the variations in peak intensity and position support the presence of both hydrogen bonding and hydrophobic effects between ChNF and TA. Additionally, electrostatic interactions likely contribute to the complexation process and should not be overlooked in certain cases.

#### 2.1.2 Structural Features

X-ray diffraction (XRD) analysis was conducted to assess the impact of TA on the crystal structure of nanochitins (Figure S3). The apparent crystallinity of ChNF was determined to be ca. 80%, while ChNC exhibited a higher value of ca. 94%. This higher crystallinity in ChNC is attributed to the selective removal of disordered regions during acid hydrolysis. Compared to ChNC, the lower crystallinity of ChNF corresponds to a looser and more irregular crystalline structure (Figure 1d), which promotes tannic acid (TA) binding through three interrelated factors:^9,11,19^ (1) Disordered molecular chains and amorphous domains form a three-dimensional network, increasing accessibility to internal binding sites; (2) Exposed free functional groups (−NH_2_and–OH) in the noncrystalline regions facilitate hydrogen bonding and electrostatic interactions with the phenolic moieties of TA; (3) A higher specific surface area maximizes the availability of reactive sites. In addition, changes in the crystallite width further reflect the interaction between TA and chitin nanofibers. As the TA content increased, the crystallite width of ChNF decreased (Figure S3), likely due to the intercalation of TA molecules into the ChNF, causing lattice distortion.^28,29^ ChNF exhibited a larger initial crystallite width than ChNC and underwent more pronounced structural changes upon interaction with TA. Collectively, these crystallinity-dependent structural and chemical features synergistically enhance ChNF–TA interfacial interactions, which underpin their superior emulsification performance. Notably, the crystallinity of the complexes decreased progressively with increasing TA content, particularly in the ChNF–TA systems.

### 2.2 Colloidal Interactions in Aqueous Suspensions

#### 2.2.1 Rheology

The rheological properties of the complexes were investigated to assess their structural behavior under shear. The apparent viscosity of the suspensions decreased with an increasing shear rate (Figure 2a_1_), a characteristic shear-thinning behavior commonly associated with the progressive breakdown of internal network structures under deformation. In the case of the ChNF bulk phase, the storage modulus (*G*′) was lower than the loss modulus (*G*″), indicating a predominantly liquid-like behavior (Figure 2a_2_). In contrast, for the ChNF–TA complexes, *G*′ exceeded *G*″, demonstrating a transition to more solid-like gel behavior. Furthermore, the difference between *G*′ and *G*″ increased with rising TA content, suggesting that TA promotes the formation of a more interconnected ChNF network through hydrogen bonding and electrostatic interactions. This behavior supports the development of a gel-like system upon complexation.^30–32^ Under an increasing shear strain, *G*′ gradually decreased and eventually fell below *G*″. The crossover point (*G′* = *G*″) marked the transition from predominantly elastic to viscous behavior, further confirming the strain-dependent structural breakdown of the network.

**Figure 2:**
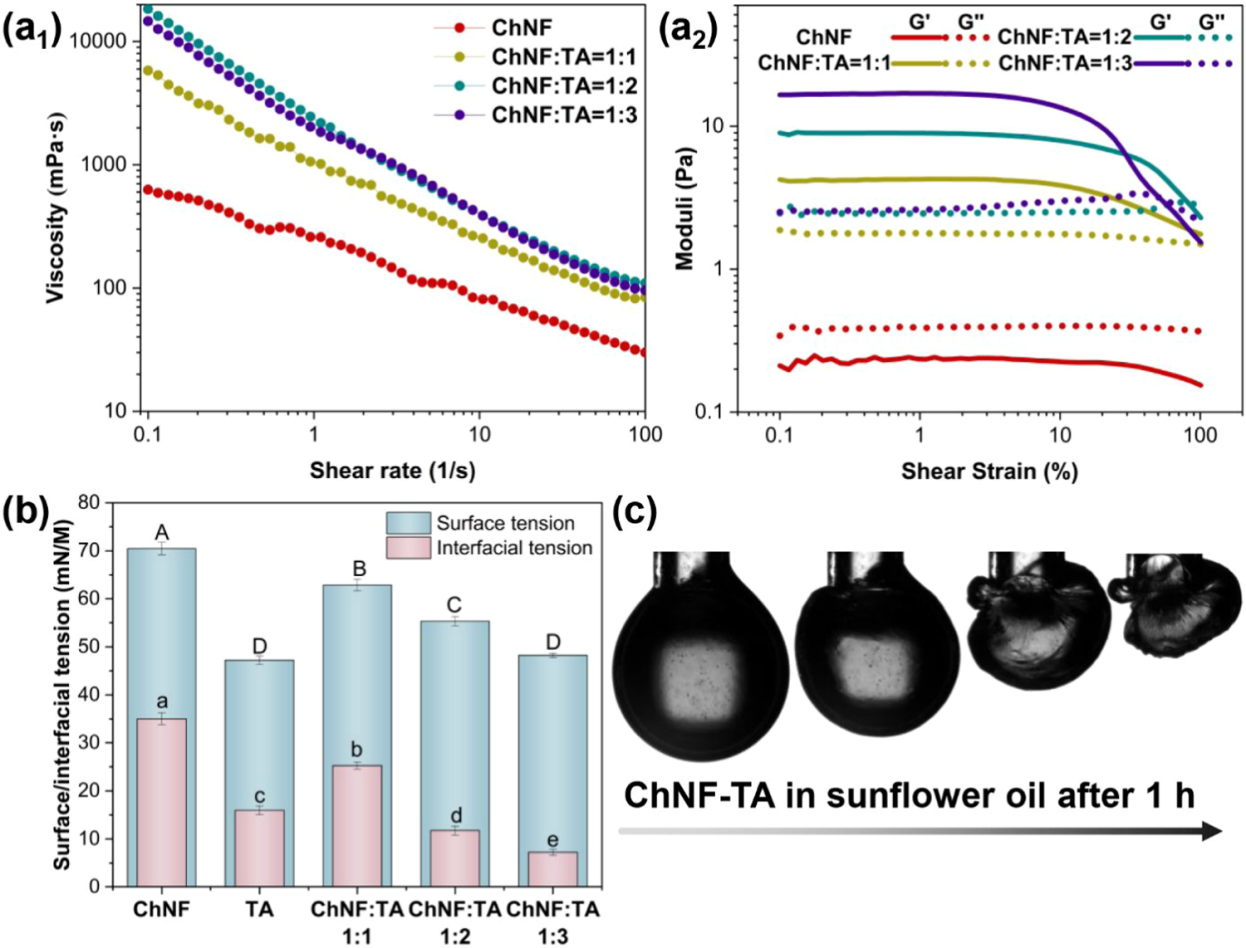
(a_1_) Flow-shear curves and (a_2_) strain sweep of aqueous suspensions of ChNF-TA complexes. (b) Corresponding surface (water/air) and interfacial (water/oil) tension of the complex suspensions. (c) Images corresponding to the morphological changes of droplets (ChNF-TA complex suspensions) immersed in the oil phase for 1 h.

#### 2.2.2 Surface and Interfacial Activity

The surface (air–water) and interfacial (sunflower oil–water) tensions of the samples were measured using the pendant drop method. As shown in Figure 2b, the addition of tannic acid (TA) effectively reduced both the surface and interfacial tensions of the aqueous suspensions.

Figure 2c illustrates the interfacial solidification of ChNF–TA complexes at the water–oil interface after 1 h. In contrast to the ChNF suspensions alone (Figure S4d), the presence of visible wrinkling on the droplets stabilized by ChNF–TA complexes points to the occurrence of interfacial complexation. This wrinkling behavior indicates the formation of a mechanically robust interfacial film with sufficient binding energy to resist deformation. The cohesive forces within the interfacial layer enabled the droplets to maintain their structural integrity, even under significant contraction and folding.

The formation of the interfacial “skin” is likely driven by strong intermolecular interactions between ChNF and TA. In particular, electrostatic attraction between the oppositely charged components facilitates the development of robust interfacial films. Similar phenomena have been reported in other biopolymer–polyphenol systems.^33^

### 2.3 Rationalization of Complexation

#### 2.3.1 Adsorption of TA on Nanochitins

To investigate the extent and dynamics of TA interaction with nanochitins, real-time monitoring of the changes in frequency (Δ*f*) and energy dissipation (Δ*D*) was conducted by using a quartz crystal microbalance (QCM-D) (Figures 3b_1_ and S5a). The adsorbed mass was determined (Figures 3b_2_ and S5b).

**Figure 3:**
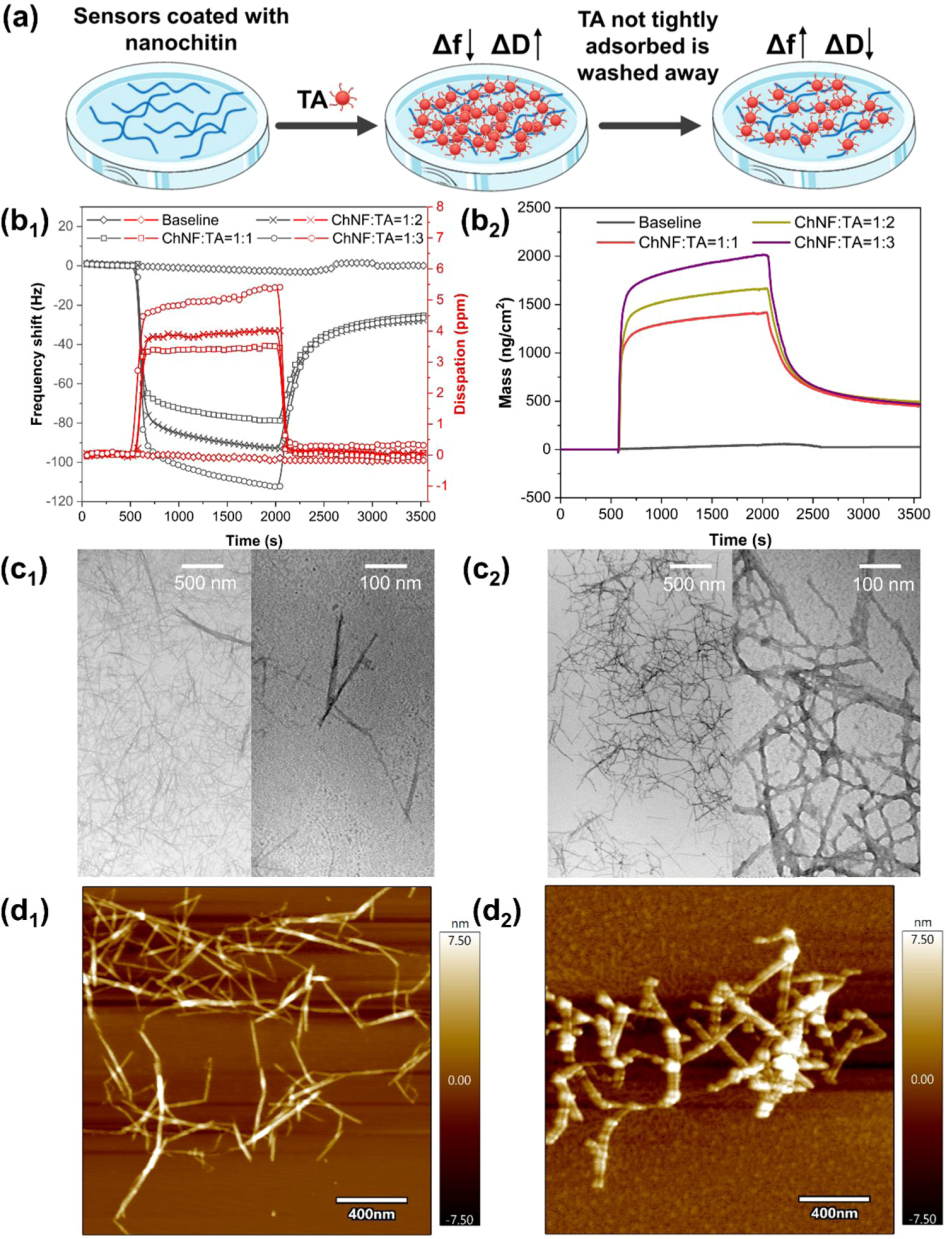
Adsorption of TA on a QCM sensor coated with ChNF. (a) Schematic illustration of the adsorption of TA on the nanochitin surface using the QCM-D. (b_1_) Frequency shift, energy dissipation, and (b_2_) apparent (QCM) adsorption amount of TA on ChNF. Microstructures observed under (c) TEM and (d) AFM images corresponding to (c_1_, d_1_) neat ChNF and (c_2_, d_2_) ChNF-TA complexes.

Initially, a TA solution was introduced over sensor surfaces uniformly coated with nanochitin. Upon exposure, a sharp decrease in Δ*f* and a simultaneous abrupt increase in Δ*D* were observed across all samples, indicating rapid adsorption of TA onto the nanochitin surfaces. These initial responses were followed by slower changes in both Δ*f* and Δ*D*, corresponding to the gradual saturation of available adsorption sites. A subsequent rinse with water led to an increase in frequency, reflecting the removal of loosely bound TA from the surface. For all measurements, the change in dissipation (Δ*D*) remained sufficiently low (Δ*D* < 6 × 10^–6^), validating the use of the Sauerbrey equation to directly calculate the adsorbed mass from the frequency shift. As the TA content increased, the frequency shifts became more negative (Δ*f*, Figure 3b_1_), while the energy dissipation (Δ*D*) increased, indicating greater adsorption and the formation of a viscoelastic adsorbed layer. Correspondingly, the adsorbed amount also increased with the TA concentration, reaching a maximum of 450 ng/cm^2^ for ChNF and 376 ng/cm^2^ for ChNC.

Microscopy analyses were conducted to confirm the QCM observations and to gain deeper insights into the morphology of the system. Transmission electron microscopy (TEM) revealed that ChNF possessed a notably high aspect ratio (Figure 3c_1_). The nanofibers were uniformly dispersed and showed no signs of agglomeration or entanglement. Following TA addition, an increase in ChNF width was observed, attributed to the adsorption of TA onto the fibril surfaces (Figure 3c_2_).

Atomic force microscopy (AFM) further supported these findings. Neat ChNF displayed a well-dispersed structure (Figure 3d_1_), while the ChNF–TA complexes exhibited a distinct knotty network morphology (Figure 3d_2_), indicative of physical cross-linking facilitated by TA

#### 2.3.2 Molecular Dynamics (MD) Simulation

To investigate the interactions between ChNF and TA at the molecular level, molecular dynamics (MD) simulations were performed at 25 °C under a constant pressure for 50 ns. The final configuration of the simulated system is shown in Figure 4a, while Figure 4b illustrates the evolution of TA distribution over time—from an initially random dispersion around ChNF to progressive adsorption onto its surface.

**Figure 4:**
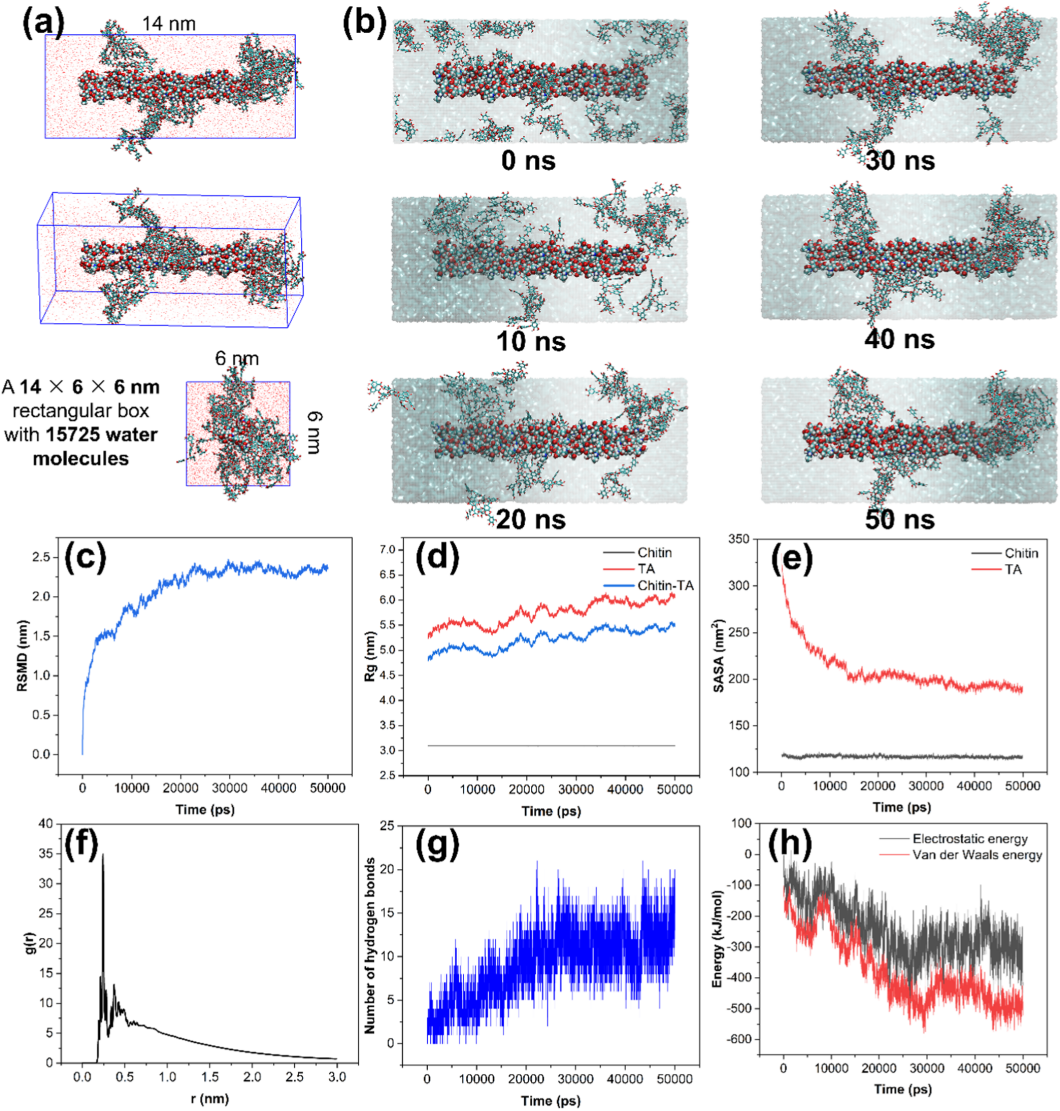
MD simulation of ChNF-TA interactions. (a) Molecular dynamics simulation boxes and (b) MD simulation conformation diagram at different times. (c) RMSD, (d) Rg, (e) SASA, (f) RDF, (g) number of hydrogen bonding, and (h) electrostatic and van der Waals energies.

The root-mean-square deviation (RMSD) values of the system remained low and stabilized after 20 ns, with a maximum fluctuation close to 2 Å, indicating that the ChNF maintained a stable and rigid structure following the interaction with TA (Figure 4c). The radius of gyration (*R*_g_) of ChNF was remained at approximately 3.1 nm, supporting its structural compactness. A difference in *R*_g_ of ∼0.8 nm between the ChNF–TA complex and free TA suggests a more compact configuration upon TA binding (Figure 4d). The solvent-accessible surface area (SASA) of ChNF exhibited minimal changes, indicating only slight molecular stretching. In contrast, TA’s SASA rapidly decreased within the first 20 ns due to its adsorption onto ChNF and possible self-association and then stabilized (Figure 4e). These observations indicate that the primary interactions between TA and ChNF were largely complete within the first 20 ns of the simulation.

Radial distribution function (RDF) analysis revealed a prominent peak at ∼0.25 nm from the center of mass of ChNF, with *g*(*r*) ∼35, suggesting a strong attraction likely driven by hydrogen bonding (Figure 4f). The number of hydrogen bonds between ChNF and TA increased over time, reaching approximately 17 by the end of the simulation, further confirming the presence of significant hydrogen bonding interactions (Figure 4g). Electrostatic and van der Waals interaction energies between ChNF and TA also increased sharply within the first 20 ns before stabilization, consistent with the RMSD and SASA trends (Figure 4h).

In summary, the MD simulations demonstrated that TA binds strongly to ChNF through a combination of hydrogen bonding, electrostatic interactions, and van der Waals forces. The resulting complexes achieved structural stability within 20 ns and remained stable throughout the 50 ns simulation period.

### 2.4 Stabilization of High Internal Phase Pickering Emulsions

Sunflower oil emulsions stabilized with ChNF and ChNF–TA complexes were evaluated for their long-term stability. The high internal phase Pickering emulsions (HIPPEs) remained stable for at least two months (Figure S6). Increasing the TA content led to a reduction in the mean droplet size, particularly in high-oil emulsions, thereby enhancing both emulsion stability and uniformity (Figure S7). Additionally, higher TA and oil contents resulted in an increased viscosity and prolonged stability (Figure S8), likely due to the formation of a stronger interfacial network and reduced fluidity (Figure 5a). To further assess the role of TA in stabilizing HIPPEs, the microstructure and rheological properties of emulsions were examined after two months of storage.

**Figure 5:**
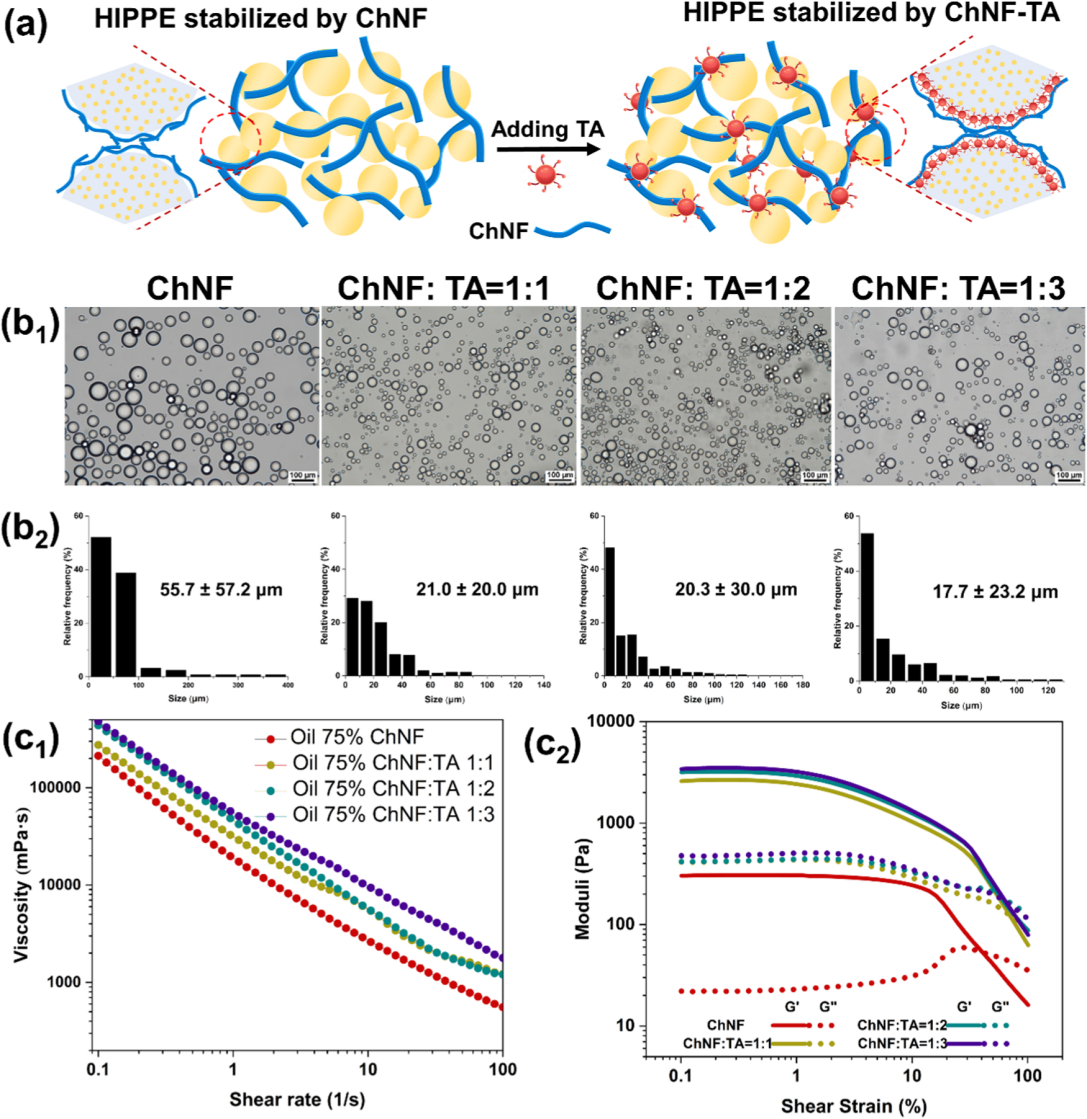
(a) Schematic illustration of the effect of TA on the stability of HIPPEs formed with ChNF. (b_1_) Micrograph and (b_2_) droplet size distributions of HIPPEs. Before taking the images, the HIPPEs were diluted 10-fold. (c_1_) Flow-shear curves and (c_2_) strain sweep of HIPPEs. HIPPEs were prepared using ChNF and ChNF-TA complexes and stored for 60 days.

#### 2.4.1 Drop Size Distribution

Figure 5b presents micrographs and droplet size distributions of HIPPEs stabilized with ChNF and ChNF–TA complexes after two months of storage. The microstructures showed no significant flocculation or aggregation, demonstrating the excellent long-term stabilization capability of the ChNF–TA complexes. Compared with HIPPEs stabilized with ChNF alone, those stabilized with ChNF–TA complexes exhibited significantly narrower droplet size distributions. Notably, the mean droplet diameter was reduced from 55 to 17.7 μm, indicating improved emulsification efficiency. This reduction in droplet size is attributed to the synergistic effect between ChNF and TA, which leads to a marked decrease in interfacial tension (Figure 2b). Lower interfacial tension minimizes droplet coalescence and reduces the energy barrier for emulsification, resulting in smaller, more uniform droplets. These effects collectively contributed to the enhanced stability of the emulsions, as further evidenced by the images captured at different storage times (Figure S6). Note: the aqueous dispersions of the individual components were colloidally stable (Figure S9).

#### 2.4.2 Rheological Properties of HIPPEs

The stability of HIPPEs is closely associated with their rheological properties, particularly the viscosity. The network structure formed by ChNF-TA in the continuous phase restricts the mobility of the emulsion droplets and inhibits phase separation. As expected, the viscosity of HIPPEs increased with rising TA content (Figure 5c_1_), which can be attributed to a decrease in droplet size and a corresponding increase in droplet number. All HIPPE samples exhibited shear-thinning behavior, as evidenced by the gradual decrease in viscosity with an increasing shear rate. This behavior likely results from the disruption of the flocculated droplet network under shear, which partially breaks down the structural integrity of the continuous phase. Additionally, shear forces may disrupt the interfacial films surrounding the droplets, promoting coalescence and increasing the effective droplet size.

Figure 5c_2_ shows that the storage modulus (*G*′) was consistently greater than the loss modulus (*G*″) within the linear viscoelastic region (0.01–10% strain range), indicating a system with predominantly elastic characteristics. Both *G*′ and *G*″, as well as the width of the linear viscoelastic region, increased with a higher TA content. These results suggest that TA enhances the structural integrity of the emulsion by promoting the formation of a more robust network. This improvement in gel strength is likely due to the progressive development of intermolecular physical cross-linking as the TA concentration increased.^34^

### 2.5 Applications of HIPPEs

#### 2.5.1 HIPPE Extrusion

Direct ink writing and 3D extrusion have gained increasing traction in the food industry due to their potential for personalized nutritional design.^35^ Given the excellent stability and viscoelasticity of HIPPEs stabilized by ChNF–TA complexes, their suitability for extrusion-based applications was evaluated. As shown in Figure 6b_1_, emulsions stabilized with ChNF alone exhibited limited self-support and quickly lost shape after extrusion. In contrast, the incorporation of TA into the ChNF phase significantly enhanced the stability of the printed structures. With an increase in TA content, the 3D print fidelity improved, as evidenced by sharper edges and better-defined geometries (Figure 6b_2,3_). These observations align with the rheological findings (Figure 5c), where TA addition enhanced viscoelasticity by reinforcing cross-linking between particle-stabilized oil droplets, thereby forming a robust gel network.

**Figure 6:**
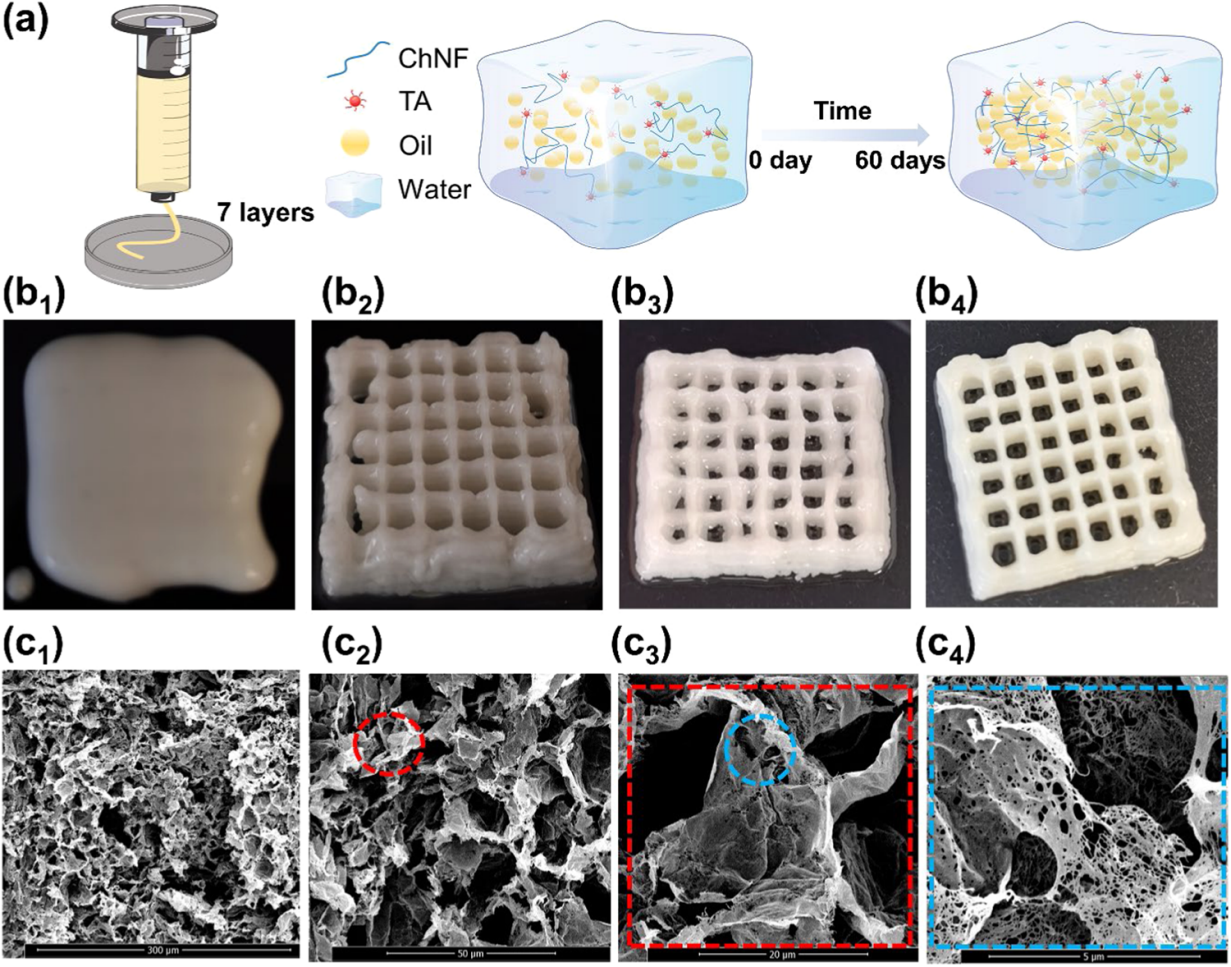
(a) Schematic illustration of the evolution of the structural components in HIPPEs over time. (b) Extruded structures were prepared with HIPPEs (seven layers). HIPPEs freshly prepared with (b_1_) ChNF, (b_2_) ChNF/TA = 1:1, and (b_3_) ChNF/TA = 1:3. (b_4_) HIPPE prepared with ChNF-TA complexes (ChNF/TA = 1:3) and stored for 60 days. (c_1_) SEM images of the porous cryogels and (c_2,3,4_) magnified SEM images of the interior of the porous cryogel. The red and blue dashed circles indicate selected locations for magnification.

Remarkably, 3D-printed structures stored at room temperature (25 °C) in the dark for 60 days retained excellent structural stability and integrity (Figure 6b_4_). This long-term preservation can be attributed to the self-supporting structure developed in the HIPPEs during storage (Figure 6a), consistent with the strong rheological behavior observed (Figure 5c). The ChNF–TA complexes, anchored firmly at the oil–water interface, formed an interconnected and highly stable interfacial network. The strong noncovalent cross-linking between TA and ChNF conferred exceptional stability in both temporal (storage) and spatial (extrusion) dimensions, making these emulsions highly promising for applications in food structuring and functional design.

#### 2.5.2 Cryogels

To gain insights into the internal architecture of the extruded emulsions, porous cryogels were synthesized by removing both the oil and the water phases. Despite the low solid content in the precursor HIPPEs, the resulting cryogels exhibited ultralow density and high porosity (Figure 6c_1_), facilitating efficient transport of both aqueous and oil phases. Importantly, the cryogels showed no signs of network collapse, indicating excellent structural integrity (Figure 6c_2,3_). This mechanical stability is attributed to the interconnectivity developed during evaporation^36,37^ and the formation of strong intra- and intermolecular interactions between ChNF and TA.^38,39^

Distinct pore sizes were observed, likely resulting from differential evaporation rates of water and cyclohexane during drying. Additionally, smaller open pores within the ChNF–TA walls may have originated from the incomplete coverage of droplet surfaces during complexation. The mechanical resilience of the cryogels is primarily derived from the inherent properties of ChNF. Detailed structural analysis (Figure 6c_4_) revealed a highly interconnected fibrillar network surrounding the pores, imparting both flexibility and elasticity to the cryogel matrix.

## 3 Conclusions

In this study, we investigated the complexation between chitin nanofibers (ChNF) and tannic acid (TA) through a combination of hydrogen bonding, electrostatic interactions, and hydrophobic effects. This resulted in viscoelastic aqueous suspensions with a reduced surface tension. The amphiphilic nature of TA enabled its effective adsorption onto ChNF, thereby modifying interfacial properties and anchoring firmly at the oil–water interface, lowering the interfacial tension at the oil–water interface, and stabilizing the corresponding O/W emulsions. The latter demonstrated exceptional stability in terms of both spatial integrity during extrusion and temporal durability during storage. The ChNF–TA complexes, formed without the need for additional reagents, present a sustainable and food-safe strategy for structuring multiphase systems. Looking ahead, the application of 3D-printed food holds particular promise for individuals with a reduced chewing ability. The possibility of tailoring food structures to meet specific nutritional and textural requirements makes this approach especially valuable for personalized nutrition- and healthcare-focused food design.

## 4 Materials and Methods

### 4.1 Materials

Chitin was purified from fresh crabs (Callinectes sapidus) obtained from Vancouver, Canada. Sunflower oil was purchased from a local supermarket and used as received. Tannic acid (TA) and gallic acid (GA) were sourced from Sigma-Aldrich, along with all other laboratory-grade reagents. Milli-Q water (18.2 MΩ·cm) was obtained using a Millipore Synergy UV system and used in all experiments.

### 4.2 Complexes Preparation

Partially deacetylated chitin and chitin nanofibers (ChNF) were prepared according to the procedure reported by Liu.^40^ When dispersed in aqueous suspension, ChNF showed a zeta potential of 61.8 ± 2 mV and particle size of 336.5 ± 0.7 nm. In an aqueous suspension, ChNF exhibited a zeta potential of 61.8 ± 2.0 mV and an average particle size of 336.5 ± 0.7 nm. Chitin nanocrystals (ChNC) were prepared following the method described by Pereira,^41^ yielding particles with a zeta potential of 34.2 ± 2.9 mV and an average size of 156.8 ± 1.2 nm.

To prepare the nanosized chitin–TA complexes, ChNF or ChNC suspensions were first prepared at a final concentration of 0.5% (w/v) by using ultrasonication. TA solutions at concentrations of 5, 10, and 15% (w/v) were added dropwise at a 10:1 volume ratio (TA solution to chitin suspension) under magnetic stirring. For the preparation of GA-based complexes, TA was replaced with GA by using the same protocol. The pH of the resulting suspensions was measured using a calibrated pH meter (FE20, Mettler-Toledo, Switzerland). All experiments were performed in triplicate.

The size and ζ-potential of the complexes were measured using a Nano Zetasizer (ZS-90, Malvern Instruments, U.K.) at 25 °C. Samples were diluted appropriately with 7.5 mM sodium chloride solution to minimize multiple scattering effects.

Ultraviolet–visible (UV–visible) transmittance spectra were recorded using a UV–Vis spectrophotometer (UV-2600, Shimadzu, Japan) with a quartz cuvette in the wavelength range of 200–400 nm. FTIR spectra were collected using a Nexus 470 FTIR spectrometer (Nicolet Instruments), averaging 32 scans per sample over the range 4000–650 cm^–1^ at a resolution of 4 cm^–1^. Data were processed using OMNIC software (Thermo Nicolet).

### 4.3 X-ray Crystallography

Freeze-dried samples were ground into fine powder and analyzed using an X-ray diffractometer (D8 Advance, Bruker, Germany). The measurements were conducted at 40 kV and 50 mA, with a scanning speed of 10°/min over a 2θ range of 5 to 70°.

### 4.4 Rheology

The viscosity and dynamic viscoelastic properties of the samples were measured using a rheometer (MCR 302, Anton Paar, Germany) equipped with a plate-and-cone geometry (1 mm gap) at 25 °C. Samples were carefully loaded onto the plate and allowed to equilibrate for 3 min to ensure thermal stability. Viscosity measurements were conducted over a shear rate range of 0.1–100 s^–1^. Dynamic frequency sweep tests were performed within the linear viscoelastic region by applying a constant strain of 1.0%, with angular frequencies ranging from 0.01 to 100 rad/s.

### 4.5 Surface and Interfacial Tensions

Surface and interfacial tensions were measured using an optical tensiometer (Attension Theta, Biolin Scientific, Finland) following the pendant drop method. For surface tension measurements, the sample was used as the dispersed phase surrounded by air, while, for interfacial tension measurements, sunflower oil served as the continuous phase. Stable droplets were formed in both cases to determine the respective tension values. All measurements were performed by using freshly prepared samples to ensure consistency and accuracy.

### 4.6 Quartz Crystal Microgravimetry

The interaction between ChNF and TA was analyzed using quartz crystal microbalance with dissipation monitoring (QCM-D) on a Q-Sense Explorer system (Biolin Scientific AB, Gothenburg, Sweden), following the experimental protocol reported by Chen et al.^42^ Gold-coated quartz crystal sensors were first cleaned by soaking in a heated (75 °C) solution of H_2_O_2_, NH_4_OH, and deionized water (1:1:5 v/v) for 10 min. After cleaning, the chips were thoroughly rinsed with deionized water and dried with nitrogen gas. ChNF suspensions were then spin-coated onto the gold sensor surface to form a uniform film.

Prior to the experiment, the QCM-D system—including inlet and outlet tubing, the measurement module, and the sensor, was rinsed with deionized water. TA solutions were injected into the flow cell at a constant flow rate of 100 μL/min, and the adsorption process was monitored in real time until a stable signal was reached. Subsequently, deionized water was introduced to remove loosely bound TA molecules.

Changes in frequency (Δ*f*) and energy dissipation (Δ*D*) were recorded at 25 °C. The adsorbed mass of TA was calculated from the frequency shift by using the Sauerbrey equation.

1 $$\Delta f=-\frac{2{f_{0}}^{2}}{A\sqrt{\rho_{q}\mu_{q}}}\Delta m$$

where Δ*f* is the change in resonant frequency of the quartz crystal, in Hz; *f*_0_ is the fundamental resonant frequency of the quartz crystal, Hz; *A* is the active area of the quartz crystal, cm^2^; ρ_q_ is the density of quartz crystal, g/cm^2^; μ_q_ is the shear modulus of quartz crystal, N/cm^2^; and Δ*m* is the change in mass on the quartz crystal surface, g.

### 4.7 Molecular Dynamics (MD) Simulations

All the all-atom MD simulations were based on a general assisted model building with energy refinement force field with the restrained electrostatic potential charges and were carried out using the Gromacs-4.6.7 software package.^43^ The system was a relaxed liquid configuration at 24.85 °C. The total run time was 50 ns under constant-pressure and constant-temperature conditions for the equilibrium MD simulation. Energy minimization was carried out with a composite protocol of steepest descent using termination gradients of 100 kJ/mol·nm. The Nose-Hoover thermostat was used to maintain the equilibrium temperature at 298 K and periodic boundary conditions were imposed on all three dimensions. The particle mesh-Ewald method was used to compute long-range electrostatics with 1 × 10^–6^ relative error.^44^ A cutoff distance of 1 nm was applied to real-space Ewald interactions. The same value was used for van der Waals interactions. The LINCS algorithm was applied to constrain the bond lengths of hydrogen atoms.^45^ A leapfrog algorithm was used with a time step of 1 fs.^46^ A 14 nm × 6 nm × 6 nm rectangular box was finally constructed, and 3 ChNF molecules (each containing 20 repeat units) were placed in the center, surrounded by 21 TA molecules, and then filled with about 15,725 water molecules.

### 4.8 Morphology

The morphology of the samples was characterized by using transmission electron microscopy (TEM; JEM-2800, JEOL, Japan) and atomic force microscopy (AFM; Nanoman VS, Bruker, Germany). For TEM analysis, a drop of the diluted suspension was deposited onto a carbon-reinforced Formvar-coated copper grid and negatively stained with a uranyl acetate solution. The sample was allowed to dry at room temperature prior to imaging, which was conducted at an acceleration voltage of 120 kV.

For AFM, the sample was diluted in water, and a droplet was deposited onto freshly cleaned mica. After the sample was dried at room temperature, imaging was performed using tapping mode at a scanning frequency of 2 Hz with a silica cantilever.

### 4.9 Emulsion Preparation

Oil-in-water emulsions were prepared by first mixing equal volumes of the aqueous suspension and sunflower oil at 22,000 rpm for 1 minute using a high-speed homogenizer (T-25 Ultra-Turrax, IKA, Germany). Subsequently, additional sunflower oil was added dropwise while homogenizing at 12,000 rpm until the desired internal phase volume fraction was achieved. The emulsions were stored at 25 °C, and their stability was assessed at intervals of 7, 14, 30, and 60 days.

Droplet size was determined by capturing emulsion micrographs using a light microscope (Nikon Eclipse LV100N POL) equipped with a 10× objective lens. Image analysis was performed with ImageJ software, where at least 200 droplets were measured to obtain the droplet size distribution. The viscosity and dynamic viscoelastic properties of the emulsions were evaluated by following the method described in Section 4.4.

### 4.10 3D Extrusion

Predesigned 3D models were fabricated using the emulsions as inks for direct ink writing (DIW)-based 3D printing. Both freshly prepared and two-month-old emulsions were used as printing materials with a BIO X printer (CELLINK, Sweden) equipped with pneumatic print heads. A 3 mL pneumatic syringe and a sterile blunt-tip needle (inner diameter: 0.51 mm) were used for all samples.

Printing was performed by using a linear fill pattern with infill densities ranging from 10 to 25%. The print head speed was set to 8 mm/s, with an extrusion speed of 0.012 mm/s, and the extrusion pressure was maintained between 20 and 40 kPa. A total of seven layers were printed at 25 °C without the use of any supporting structures. All 3D-printed HIPPE structures were stored at room temperature (25 °C), protected from light, and monitored over time for morphological changes.

### 4.11 Cryogels

Cryogels were prepared using emulsions as templates, substituting sunflower oil with cyclohexane at a final internal phase volume fraction of 75%.^47^ The resulting samples were freeze-dried, and their micromorphology was examined using scanning electron microscopy (SEM; FEI Helios Nanolab 650, Netherlands) at an accelerating voltage of 10 kV.

## Supplementary Material



## Notes

Experimental data for pure components (ChNF, ChNC, TA, GC) and the complexes (ChNF-TA, ChNF-GA, ChNC-GA and ChNC-GA): UV and FTIR spectra to elucidate main chemical changes; pH, ζ-potential and particle size; XRD profiles; surface and interfacial activity; rheological properties and changes observed on the surface of droplets (ChNF suspensions) immersed in the oil phase; QCM frequency and energy dissipation shift along with adsorption amount of TA on ChNC; emulsion stability data; emulsion droplet size; rheological properties of emulsions stored for 60 days; and images of the ChNF and ChNF-TA complex suspensions (PDF)
