# Supplementary material for: Interfacial Stabilization of Green and Food-Safe Emulsions through Complexation of Tannic Acid and Nanochitins
Source: ACS Appl Mater Interfaces. 2025 May 31;17(23):34647–58. doi: 10.1021/acsami.5c00132 (PMC12163926; doi:10.1021/acsami.5c00132)
Supplement: Supplementary file 1 [file am5c00132_si_001.pdf]

Supporting Information

Interfacial stabilization of green and food-safe emulsions through complexation of tannic acid and nanochitins

Qian Wu[a,b], Chen Zhou[a], Mengyao Niu[a], Jiaxin Hu[c], Nianjie Feng[b,c,], Sameer E. Mhatre[b], Yi Lu[b], Xun Niu[b], Tianyu Guo[b], Jingqian Chen[b], Ran Bi[b,*], Orlando J. Rojas[b,d,e,f,*]*

[a] Hubei Key Laboratory of Industrial Microbiology, Key Laboratory of Fermentation Engineering (Ministry of Education), National "111" Center for Cellular Regulation and Molecular Pharmaceutics, Hubei University of Technology, Wuhan, 430068, Hubei, P.R. China

[b] Bioproducts Institute, Department of Chemical & Biological Engineering, The University of British Columbia, Vancouver, V6T 1Z3, BC Canada.

[c] School of Material Science & Chemical Engineering, Hubei University of Technology, Wuhan, Hubei, 430068, China.

[d] Department of Chemistry, 2036 Main Mall. Vancouver, The University of British Columbia, Vancouver, V6T 1Z1, BC Canada.

[e] Department of Wood Science, The University of British Columbia, 2900-2424 Main Mall Vancouver, V6T 1Z4, BC Canada.

[f] Department of Bioproducts and Biosystems, School of Chemical Engineering, Aalto University, FI-00076 Aalto, Espoo, Finland.

Corresponding author: Orlando J. Rojas (orlando.rojas@ubc.ca; orlando.rojas@aalto.fi)

This supporting Information Document contains 9 figures in 11 pages.

Abbreviations: ChNF=chitin nanofibrils, ChNC=chitin nanocrystals, GA= gallic acid, TA=tannic acid.

UV-Vis spectra. The UV-Vis spectra of the complexes in the wavelength range of 200 to 400 nm are given in Figure S1a. All complexes exhibit a hydroxyl absorption peak near 230 nm and a benzene ring absorption peak near 275 nm. Nanochitins showed no major absorption peak between 200 to 400 nm. The absorbance of the complexes increased with increasing tannic acid (TA) of gallic acid (GA) content and the spectral wavelengths exhibited a slight red shift due to the presence of chromophores.

FTIR spectra. The peaks at 3443 cm^{-1} and 3260 cm^{-1} of ChNF correspond to the O-H and N-H stretching vibrations, respectively. The peak at 2874 cm^{-1} in ChNF is associated with the CH stretching vibration. The peak at 1655 cm^{-1} of ChNF corresponds to amide group C-O stretch (amide I band). The N-H bond of the acetamide (amide II band) is at 1554 cm^{-1}. The peaks assigned to the C-O group stretching vibration is at 1068 cm^{-1}. Similar peaks assignment were observed in the FTIR spectra of ChNC (Figure S1b_2 and S1b_3).

The characteristic peaks of GA at 3493 cm^{-1} and 3272 cm^{-1} correspond to stretching modes of the different O-H groups (Figure S1b_1 and S1b_2). The stretching and bending vibrations of aromatic ring C-C/C-H were mainly characterized by bands in the range of 1607-1422 cm^{-1}. The bending vibrations of C-H and O-H in the ring were in the range of 1309-1217 cm^{-1}. The stretching and bending vibrations of C-O groups were in the range of 1022-731 cm^{-1}.

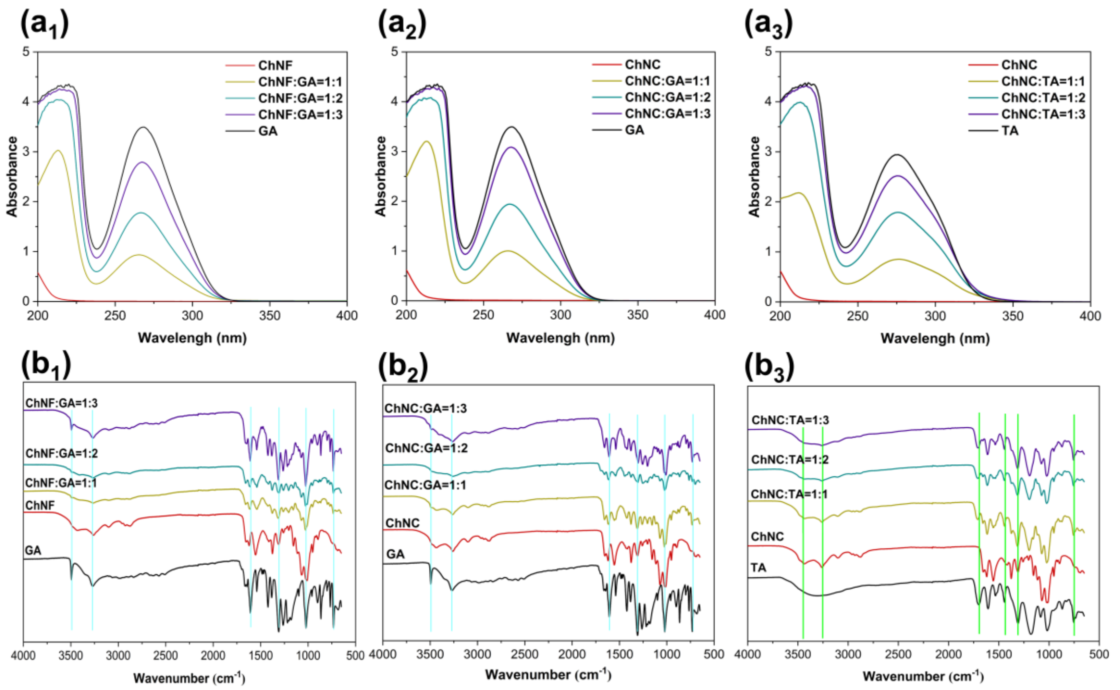

Figure S1. (a) UV-Vis and (b) FTIR spectra of the complexes: (a_1, b_1), ChNF-GA (a_2, b_2) ChNC-GA, and (a_3, b_3) ChNC-TA. ChNF=chitin nanofibrils, ChNC=chitin nanocrystals, GA= gallic acid, TA=tannic acid.

pH, ζ-potential and particle size. Here we show the changes in the pH of nanochitin suspensions after the addition of TA or GA. As shown in Figure S2a, the pH decreased with the addition of TA or GA and was lower than the pKa of nanochitins. The absolute values of ζ-potential of the complexes were all greater than 20 (Figure S2b), indicating good colloidal stability. The ζ-potentials of ChNF-TA and ChNF-GA complexes decreased significantly with the TA or GA content, especially at high concentrations of the latter components. In contrast, there was no significant reduction of ζ-potentials in ChNC suspensions, which indicated that TA/GA interacted less strongly with ChNC than with ChNF. A lower surface charge was measured for ChNC, likely related to the low and inhomogeneous distribution of amino groups[1], resulting in

the weak interaction with TA/GA. The particle size of ChNF was larger than that of ChNC (Figure S2c), consistent with previous studies[2]. Unlike the ChNF-TA complexes, there was no significant difference in the particle size of other complexes.

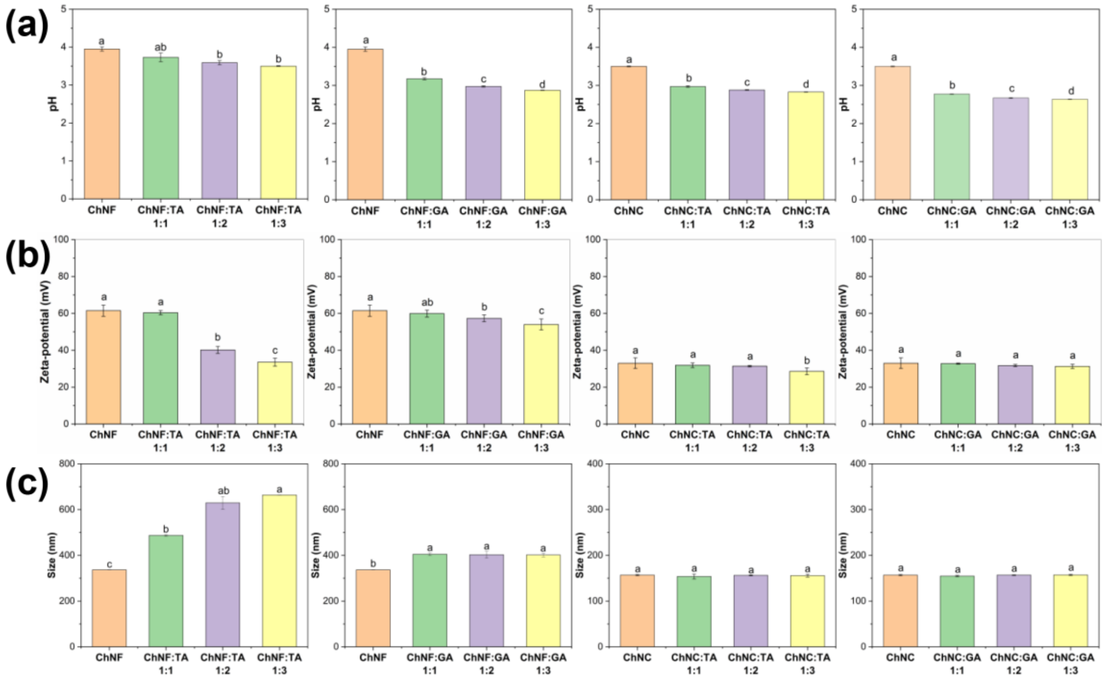

Figure S2. The (a) pH, (b) ζ-potential and (c) particle size of the complexes.

XRD profiles. Nanochitins had a strong diffraction peak at 9.2°and 19.4° (Figure S3). The crystallinity of ChNF was 79.9%, while that of ChNC was 93.9%. This is attributed to the removal of disordered regions during acid hydrolysis. Broader diffraction peaks of TA are present at 13.8° and 25.2°. Various notable peaks were identified for GA at diffraction angles 2θ of 16.0°, 24.7°, 26.2° and 28.0°. As TA/GA content increased, the crystallinity of the complexes decreased.

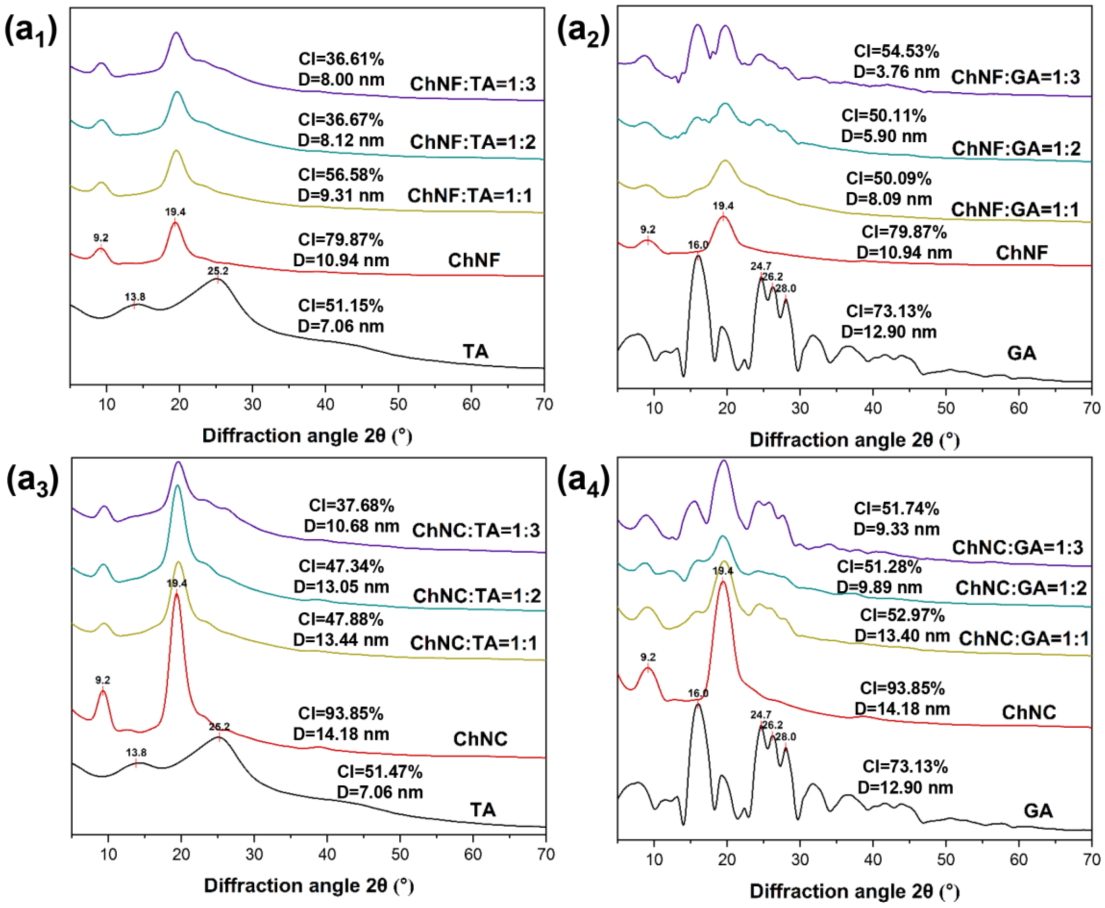

Figure S3. XRD profiles corresponding to complexes formed by (a) ChNF-TA, (b) ChNF-GA, (c) ChNC-GA and (d) ChNC-GA. CI is the crystallinity index and D is the crystallite width.

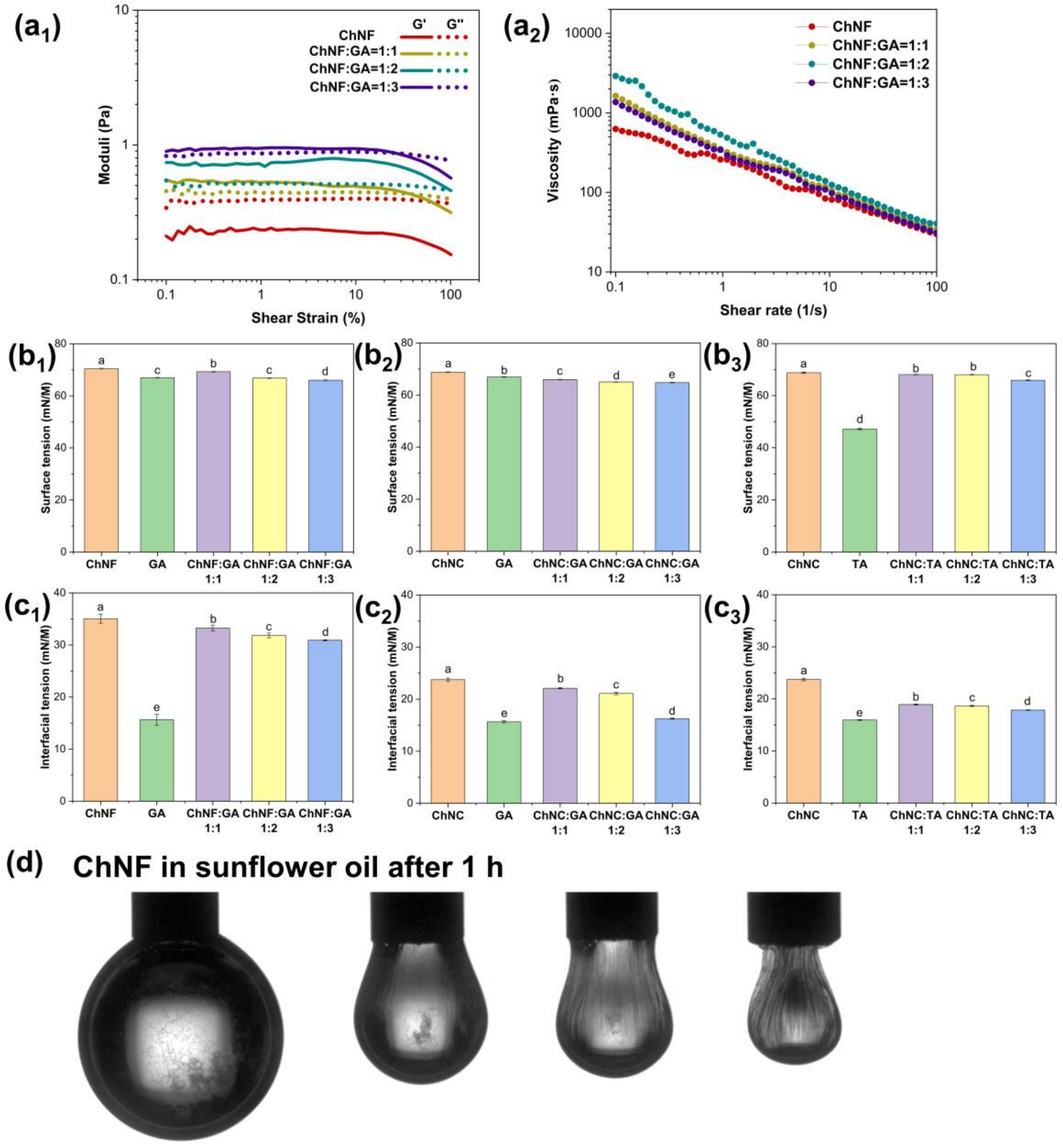

Figure S4. Surface and interfacial activity of the complexes. (a) Rheological properties of the ChNF-GA complexes. (b) Surface and (c) interfacial properties of ChNF-GA, ChNC-GA and ChNC-TA complexes. (d) Images showing changes in droplets of ChNF suspensions immersed in the oil phase after 1-hour.

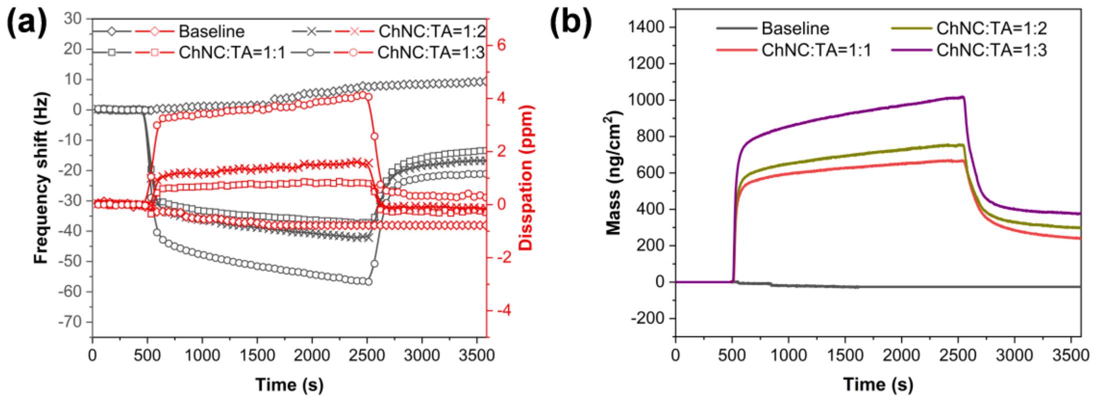

Figure S5. (a) QCM frequency energy dissipation shift. (b) Corresponding Sauerbrey mass of TA adsorbed on ChNC.

Stability of emulsions. The emulsions were observed during give time periods. After 7 and 14 days of storage, the emulsions were stable, as presented in Figure S6b and S6c. After one month of storage, only the high viscosity emulsions remained stable (Figure S6d). At two months of storage, only emulsions with over 60% oil content remained stable (Figure S6e). The results suggest that the ChNF-TA complexes stabilized HIPPEs for a long time.

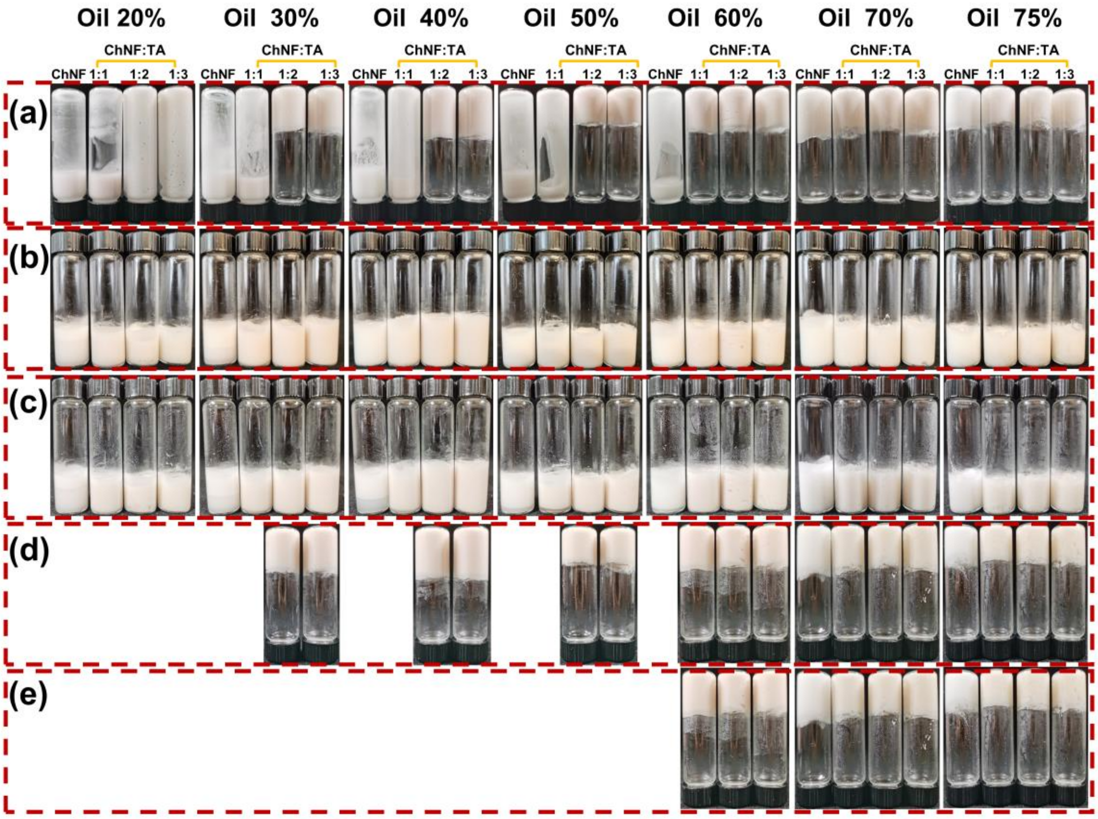

Figure S6. Stability of emulsions at different time periods. Images from left to right show emulsions with given oil-water ratios (20-75%), where the emulsions were stabilized by the ChNF and ChNF-TA complexes (1:1, 1:2 and 1:3): (a) freshly prepared emulsions; (b) emulsions stored for 7 days, (c) 14 days, (d) 30 days and (e) 60 days.

Droplet size distribution. The mean droplet size of the ChNF-based emulsions was rapidly reduced by the addition of TA, and the sizes continued to decrease with the increase of TA content (Figure S7). The sizes ultimately fell within the range of 13-20 μm.

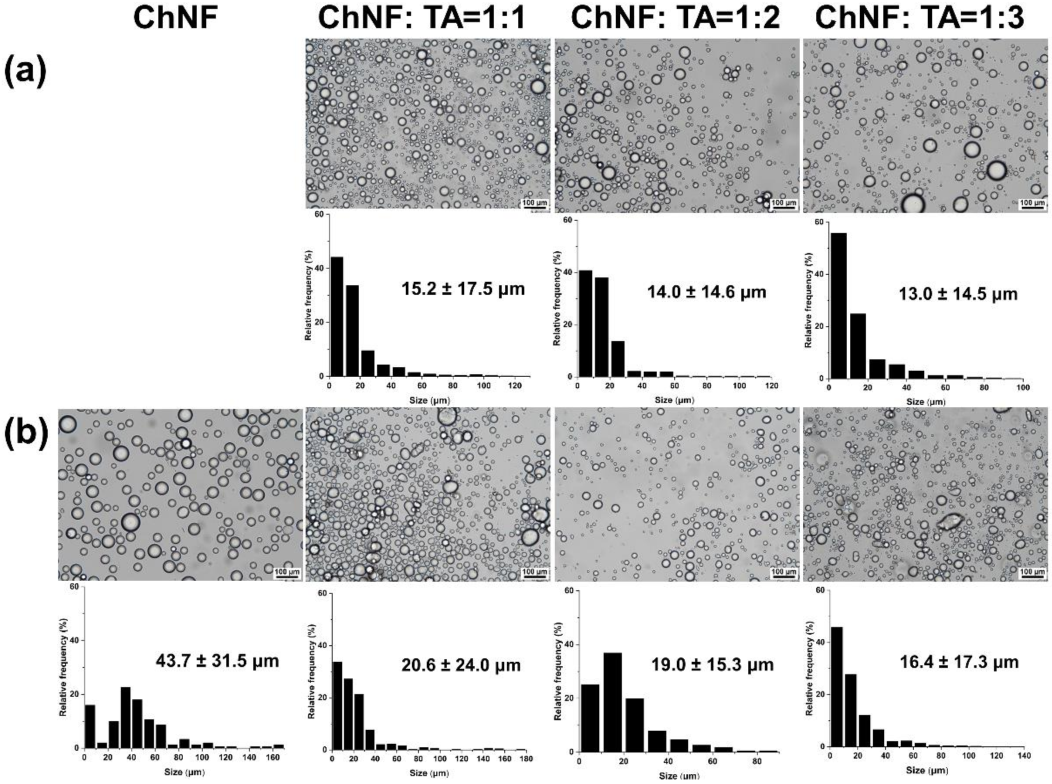

Figure S7. Micrographs and droplet size distribution of ChNF-TA complexes stabilized oil-in-water emulsions. The emulsions with different oil/water ratio (60% for a, 70 for b) were prepared at different ratios of ChNF and TA (1:1, 1:2 and 1:3). Droplet size distributions were analyzed using Image J software.

Rheological properties. The emulsions with over 60% oil content had higher G' and G″ (Figure S8), indicating that the increase of oil content made the emulsions more viscoelastic. The strength of the complexes was enhanced with the increase of TA content. A more robust and stable gel network structure was produced.

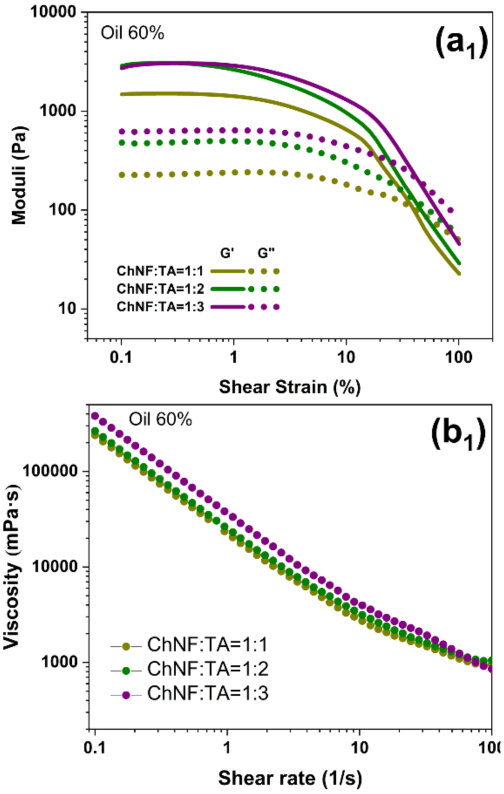

Figure S8. Rheological profiles of emulsions stored for 60 days. (a) strain sweep, (b) flow-shear curves.

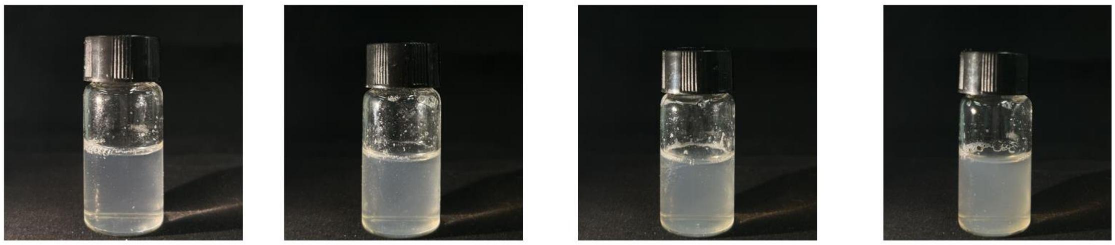

ChNF ChNF:TA=1:1 ChNF:TA=1:2 ChNF:TA=1:3

Figure S9. Images of aqueous dispersions of ChNF and ChNF-TA complexes.

References

(1) Lu, Y.; Kamkar, M.; Guo, S.; Niu, X.; Wan, Z.; Xu, J.; Su, X.; Fan, Y.; Bai, L.; Rojas, O. J. Super-Macroporous Lightweight Materials Templated from Bicontinuous Intra-Phase Jammed Emulsion Gels Based on Nanochitin. *Small.* **2023**, *19* (39), 2370315.

(2) Zhang, M.; Li, Y.; Wang, W.; Yang, Y.; Shi, X.; Sun, M.; Hao, Y.; Li, Y. Comparison of Physicochemical and Rheology Properties of Shiitake Stipes-Derived Chitin Nanocrystals and Nanofibers. *Carbohydr. Polym.* **2020**, *244*, 116468.
